# Increased Aggregation Is More Frequently Associated to Human Disease-Associated Mutations Than to Neutral Polymorphisms

**DOI:** 10.1371/journal.pcbi.1004374

**Published:** 2015-09-04

**Authors:** Greet De Baets, Loic Van Doorn, Frederic Rousseau, Joost Schymkowitz

**Affiliations:** 1 VIB Switch Laboratory, Flanders Institute for Biotechnology (VIB), Leuven, Belgium; 2 Switch Laboratory, Department of Cellular and Molecular Medicine, University of Leuven, Leuven, Belgium; Fudan University, CHINA

## Abstract

Protein aggregation is a hallmark of over 30 human pathologies. In these diseases, the aggregation of one or a few specific proteins is often toxic, leading to cellular degeneration and/or organ disruption in addition to the loss-of-function resulting from protein misfolding. Although the pathophysiological consequences of these diseases are overt, the molecular dysregulations leading to aggregate toxicity are still unclear and appear to be diverse and multifactorial. The molecular mechanisms of protein aggregation and therefore the biophysical parameters favoring protein aggregation are better understood. Here we perform an *in silico* survey of the impact of human sequence variation on the aggregation propensity of human proteins. We find that disease-associated variations are statistically significantly enriched in mutations that increase the aggregation potential of human proteins when compared to neutral sequence variations. These findings suggest that protein aggregation might have a broader impact on human disease than generally assumed and that beyond loss-of-function, the aggregation of mutant proteins involved in cancer, immune disorders or inflammation could potentially further contribute to disease by additional burden on cellular protein homeostasis.

## Author Summary

Protein aggregation has been recognized to contribute to the development of more than 30 human diseases such as Alzheimer and Parkinson disease. Here we have performed an *in silico* survey of human sequence variations to evaluate whether protein aggregation might impact human disease beyond the above-mentioned aggregation diseases. We find that human disease mutations are more likely to increase the aggregation potential of proteins than non-disease associated mutations. This survey therefore suggests the possibility that protein aggregation is a more widespread disease modifier than previously expected.

## Introduction

Protein aggregation is found to be associated to an increasing number of human diseases [1]. In many cases aggregation directly contributes to or modulates the pathology with which it is associated. The mode of action of these protein aggregates in disease is generally classified into loss-of-function and gain-of-function effects [2]. Loss-of-function results from the sequestration of misfolded proteins into inactive cellular inclusions and can functionally be equated to a genetic deletion. In addition, aggregated proteins can also acquire novel aggregation-specific functions that further contribute to the disease. In this case, the presence of an aggregated protein results in a worse disease outcome than the absence of the native protein. In Alzheimer disease for example, Aβ peptide aggregation generates synaptotoxic activity leading to neurodegeneration, while absence of the Aβ peptide does not result in neuronal loss. However, the mechanisms whereby protein aggregates acquire gain-of-function in more than 30 neurodegenerative diseases remain largely unknown. *In vitro* evidence showing that small amyloid-like aggregates perforate biological membranes supports the assumption that protein aggregates act as lethal toxins and that these properties emanate from generic structural properties of amyloid aggregates [3]. Recent evidence however suggests that (1) gain-of-function is not restricted to amyloid aggregates and (2) aggregates can acquire alternative gain-of-function activities that are not directly cytocidal but rather modify cell physiology in more subtle ways. For instance, it was found that non-amyloid aggregation of p53 confers oncogenic gain-of-function activity to tumors resulting in increased cell proliferation rather than apoptosis [4]. In familial Fabry disease, an archetypical loss-of-function disease resulting from α-galactosidase inactivation, aggregating mutants nevertheless acquire gain-of-function in the form of pharmacological resistance to the chemical chaperone DGJ-1[5].

These results suggest that neurodegenerative and other amyloid diseases only form the tip of the iceberg and that protein aggregation might be implicated in far more pathologies than presently suspected, including cancer and metabolic diseases. In order to probe the potential of protein aggregation as a disease-modifying factor, we here analyze a curated set of polymorphisms and disease-associated mutations from a VariBench subset[6] for which structural information is available (5480 pathogenic and 1015 neutral mutations).

**Fig 1:**
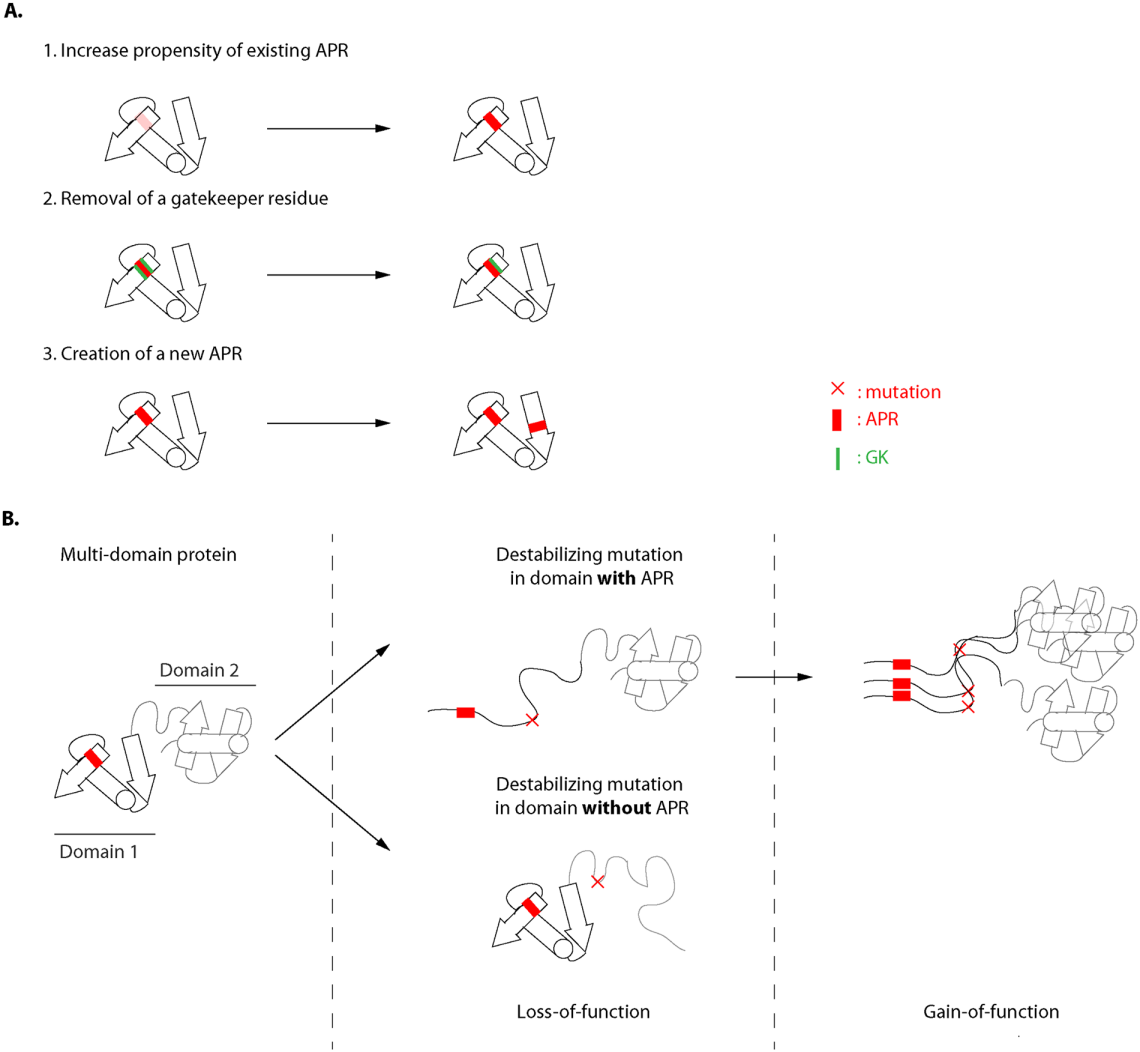
Schematic overview of how mutations can increase the aggregation tendency of a protein. A) Mutation increasing the intrinsic aggregation tendency by 1) increasing the aggregation propensity of an existing APR, 2) removal of a gatekeeper residue, or 3) introduction of a new APR in the protein. B) Mutation increasing the aggregation tendency by destabilizing the protein and exposing the APR to the environment. We assume that destabilizing mutations are more likely to expose APRs present in the comprising domain than in the neighboring domains.

Protein aggregation is determined by short aggregation prone regions (APRs) that are generally buried in the hydrophobic core of the protein where they participate in the stabilization of tertiary interactions. However, when proteins get (partially) unfolded, these APRs become solvent exposed and can self-assemble into aggregates by forming intermolecular β-strand interactions (Fig 1A) [7–9]. The aggregation potential of a protein is thus determined by two factors: 1) the tendency of APRs to self-assemble by β-strand aggregation (i.e. the intrinsic aggregation propensity of the polypeptide sequence) and 2) the availability of these APRs as determined by the stability of the native protein. Mutations that increase the intrinsic aggregation of a protein sequence, destabilize its protein structure or both, will increase the potential for aggregation of a given protein.

The effect of these mutations on protein aggregation is evaluated with a set of computational tools calculating the intrinsic aggregation propensity of the unfolded protein chain (TANGO[10]) as well as the thermodynamic effect of mutations on the stability of the native protein (FoldX[11]). Mapping the conjugated effect of these two aggregation-determining parameters on mutated protein domains rather than on full-length proteins, we here identify a characteristic signature of aggregation enhancing human variations and find that 22,5% of disease mutants in the VariBench set result in enhanced aggregation propensity in comparison to 7,5% in human polymorphisms. Our results suggest that aggregation might be a disease modifier in a wide range of human diseases including metabolic diseases, infection and immunity, and especially cancer. Given the high incidence of aggregation-promoting mutations in cancer we further compared the COSMIC database[12] with the 1000 genomes dataset[13]. This confirmed the enrichment of aggregation prone mutants in cancer mutations, as 38% of cancer mutations result in an increased aggregation propensity of the affected protein.

## Results

### Pathogenic variants are enriched in mutants that increase the aggregation propensity of globular protein sequences

To analyze the effect of disease-associated and neutral mutations on protein aggregation, an unbiased and representative benchmark dataset is required. VariBench[6] overcomes this problem and offers datasets of experimentally verified high-quality data, either from literature or relevant databases. More specifically, the neutral dataset, comprising 21,170 human non synonymous coding SNPs, and the pathogenic dataset, comprising 19,335 mutations, were selected.

The intrinsic aggregation propensity of a protein is defined as the propensity of an unfolded protein sequence to aggregate. Independent grafting experiments have shown that the intrinsic aggregation propensity is related to the presence of short aggregation-prone regions (APR) that self-associate to form intermolecular β-structured assemblies. These APRs are typically short sequence segments (5–15 amino acids) that display high hydrophobicity, low net charge, and a high tendency to form β-structures[14]. A variety of methods have been developed to identify such APRs in amino acid sequences[15,16] and in this study the TANGO algorithm[10] was used. TANGO is a statistical thermodynamics algorithm that identifies aggregation nucleation sites by not only considering the factors described above, but also the competition between β-sheet formation and other structured states.

A proteome-wide analysis using TANGO has shown that 10.6% of the residues in the entire human proteome are part of an APR (1168232 APR residues over 11071210 amino acids) and thus directly contribute to the intrinsic aggregation propensity of the unfolded polypeptide chain. We find that the frequency of mutations falling within APRs is random and amounts to 11,3% in neutral mutations whereas this is enriched to 15,4% in disease mutants (p<0.00001, Chi- square test), as such modifying the intrinsic aggregation tendency. The aggregation propensity of an APR can also be modified by mutations in so-called gatekeeper residues, i.e. residues that directly flank APRs (positions -3 and +3) and the role of which is to slow aggregation kinetics and mediate chaperone interactions [17,18]. Gatekeeper residues generally consist of charged residues (Arg, Lys, Glu, Asp) and proline that counteract aggregation by i) charge repulsion (Arg, Lys, Glu, Asp); ii) being large and flexible (Arg and Lys); or iii) being incompatible with the beta-structure (Pro and Gly)[19,20]. A proteome-wide study has shown that 90% of all APRs are capped with at least one gatekeeper residue[20]. Consistent with their role in controlling protein aggregation, we found in the dataset analyzed here that 12% of the pathogenic mutations affect these gatekeeper residues, versus 8% of the neutral mutations (p<0.00001, Chi- square test).

Filtering out only the mutations that increase the intrinsic aggregation and discarding those that reduce or do not affect the intrinsic aggregation propensity of APRs, 40.8% of pathogenic mutations affecting the APR or the surrounding gatekeepers actually increase the intrinsic aggregation tendency and an additional 4.3% of the pathogenic mutations increase the intrinsic aggregation propensity by causing *de novo* creation of an APR that is not present in the wild type sequence (only 1.7% for neutral mutations, p<0.00001, Chi- square test). To summarize, pathogenic mutations seem to increase the intrinsic aggregation propensity more often than neutral mutations, respectively 15.5% and 10.1% (p<0.00001, Chi- square test). This can occur either through 1) increasing the aggregation propensity of an existing APR, 2) removal of a gatekeeper residue, or 3) introduction of a new APR in the protein.

### Aggregation-promoting pathogenic variants are not enriched in disordered protein sequences

In order to estimate the impact of aggregation-promoting variants on unstructured proteins, we identified all unstructured protein segments in the entire VARIBENCH set using the IUPRED algorithm [21]. This analysis revealed that 12% and 24% of respectively pathogenic and neutral variants are within unstructured protein domains (9.1% and 18.4% of disordered residues in pathogenic and neutral set). Variants within unstructured protein domains only marginally affect the intrinsic aggregation propensity of the amino acid sequence and are not significantly enriched in pathogenic variants (1.9% and 1.4% in pathogenic and neutral mutants respectively increase the intrinsic aggregation propensity of unstructured protein sequences (p>0.05, Chi- square test)). This observation is not unexpected: as the sequence composition of unstructured protein sequences are enriched in charged and polar residues and therefore have a lower hydrophobic content, the frequency of APRs in unstructured protein domain sequences is approximately three times lower than in globular domains [22] reducing the probability of mutations that increase the propensity of APRs. Moreover, the low hydrophobic moment of these sequences also makes *de novo* creation of APR by a single mutation much more unlikely. Finally, as these domains are devoid of tertiary structure the increase of aggregation by exposing APRs through structural destabilization are *de facto* absent. We therefore conclude that disease mutations are less likely to induce protein aggregation in unstructured protein domains than in globular protein domains. However, this does not mean that aggregation is irrelevant for unstructured proteins. Indeed, important proteopathies such as Parkinson disease (alpha-synuclein) and amyotrophic lateral sclerosis (TDP-43, Fus) are associated with the aggregation of unstructured proteins.

### Pathogenic variants are enriched in mutations that expose aggregation-prone regions through destabilization of the native state

The previous sections describe the effect of mutation on the intrinsic aggregation propensity, *i*.*e*. the aggregation propensity of the unfolded protein. However, under native condition, the APRs that define the intrinsic aggregation tendency are often ‘protected’, i.e. they are generally unavailable for aggregation as they participate in the network of contacts that stabilize the native state [23–26]. However, mutants that thermodynamically destabilize the native state or at least the structural region in which an APR is embedded will result in an increased likelihood that this APR is unfolded and solvent exposed, and thus available for self-assembly into β-structured aggregates. The role of protein destabilization in aggregation-associated human diseases has been amply documented [1] and this is e.g. the case for familial mutations in transthyretin (TTR) [27] and lysozyme [28]. In these proteins, mutations affecting the protein stability expose an APR that drives aggregation.

**Fig 2:**
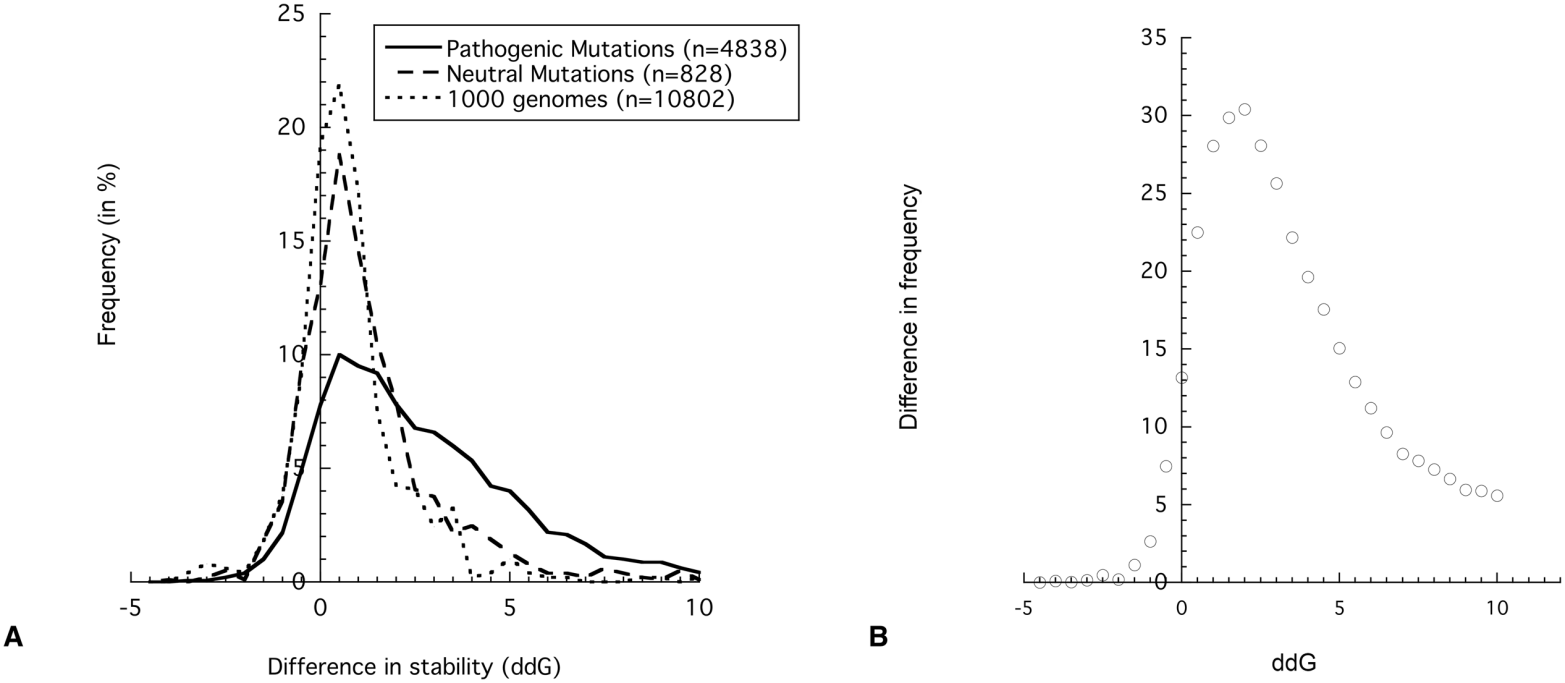
Effect of mutations on protein stability. A) Histogram for the efffect of stability (ΔΔG) for i) the pathogenic dataset, ii) the neutral dataset and iii) SNPs identified in the 1000 genomes project. A negative ΔΔG indicates a stabilizing mutation, whereas a positive ΔΔG indicates that the mutation disturbs the protein stability. B) Enrichment of pathogenic mutations based on ΔΔG. The difference in the frequency of pathogenic mutation and neutral mutations is plotted for each ΔΔG interval, where a positive value indicates an enrichment of disease mutations.

To assess the effect of mutation on the thermodynamic stability of APRs in our VARIBENCH dataset, the FoldX forcefield[11] was used. This empiric forcefield allows obtaining a fast and accurate estimation of the free energy change of protein stability upon mutation (called ΔΔG, expressed in kcal/mol), starting from a high-quality crystallographic structure. Therefore, the VARIBENCH set used above has been filtered for variations in proteins having either an experimentally determined crystal structure in the Protein Data Bank (PDB) [29], or a high-quality homology model (homology ≥ 90). This filtering resulted in a final dataset of 5480 pathogenic and 1015 neutral mutations. On this set, we confirmed the previously known observation that pathogenic mutations are generally more destabilizing than neutral mutations[30,31] (p = 1 x 10^−66^, Mann-Whitney U test) (Fig 2A): using a threshold of ΔΔG > = 2 kcal/mol results in an enrichment of 30% of destabilizing variants in disease-associated mutations (Fig 2B).

As severe structural destabilization generally results in loss-of-function by disruption of binding and catalytic sites, this explains why thermodynamic destabilization is more frequent in pathogenic mutations. However, as discussed above, thermodynamic destabilization will also result in the solvent exposure of APRs in misfolded proteins resulting in an increased aggregation propensity of disease mutants, a factor that can potentially contribute to additional pathophysiological stresses.

### Pathogenic variants are enriched in mutations that affect protein domains with strong APRs

The majority of proteins are composed of multiple structural domains that fold more or less independently. As a result, the structural consequences of a destabilizing mutation will be most severe for the structural domain in which the mutant is located even though its effect will generally not be restricted to it. Consequently, destabilizing mutations are more likely to expose APRs present in the comprising domain than in neighboring domains (Fig 1B). To gain further evidence that aggregation plays an important role beyond loss-of-function in shaping the pathogenic nature of disease mutants, we compared the enrichment of destabilizing mutations in whole proteins with the enrichment of destabilizing mutations in the individual protein domains. If aggregation plays a role in the pathogenic nature of mutations, there should be a stronger enrichment of destabilizing mutations in domains that possess strong APRs.

**Fig 3:**
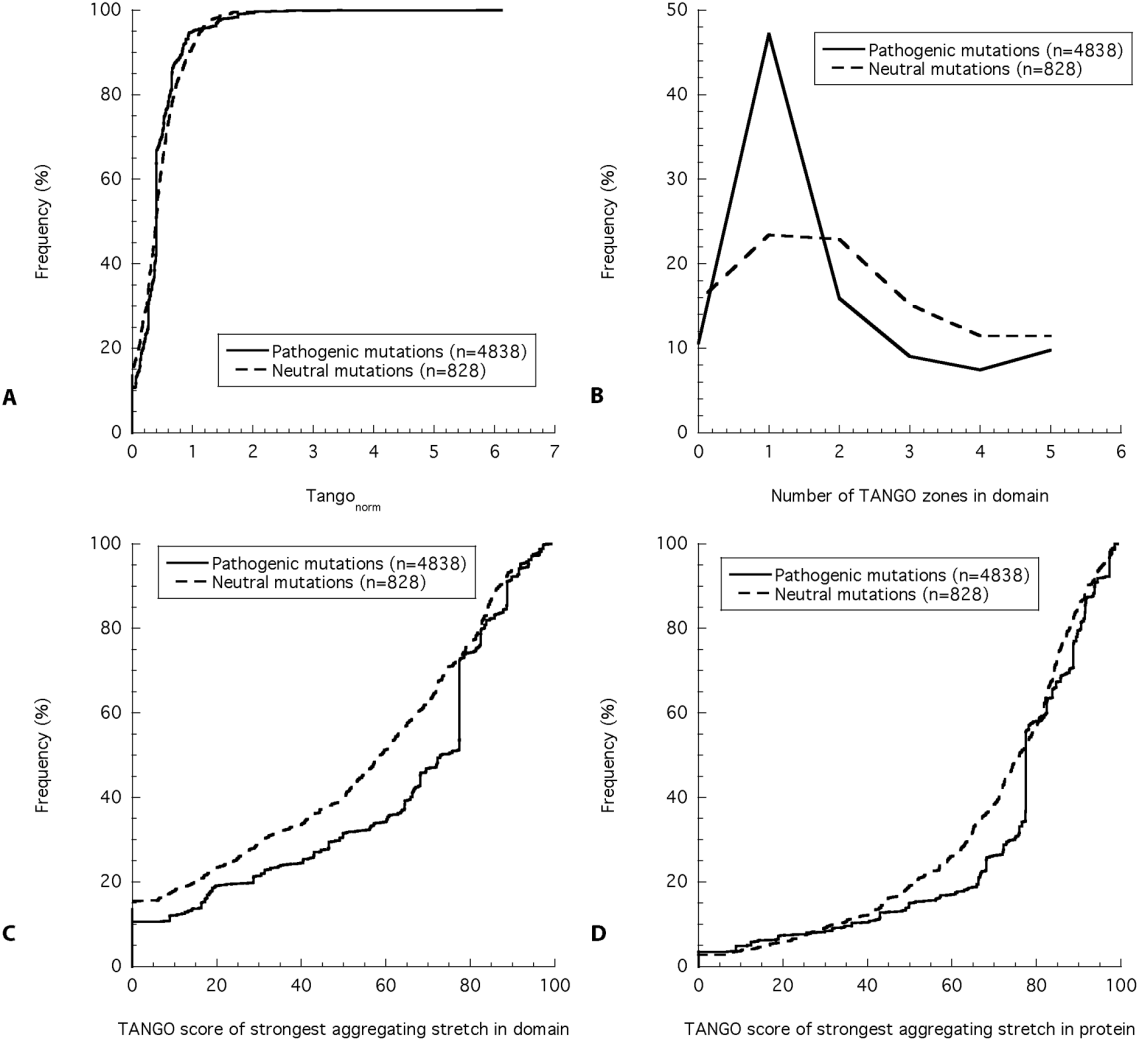
Analysis of the aggregation propensity in structural domains. The A) normalized TANGO score, B) number of aggregating stretches, and C) score of the strongest aggregating stretch in the structural domain with the non-synonymous mutation (pathogenic or neutral) represented as cumulative frequency (A, C) or frequency (B) plot. (D) Cumulative frequency plot of the score of the strongest aggregating stretch in the complete protein.

**Fig 4:**
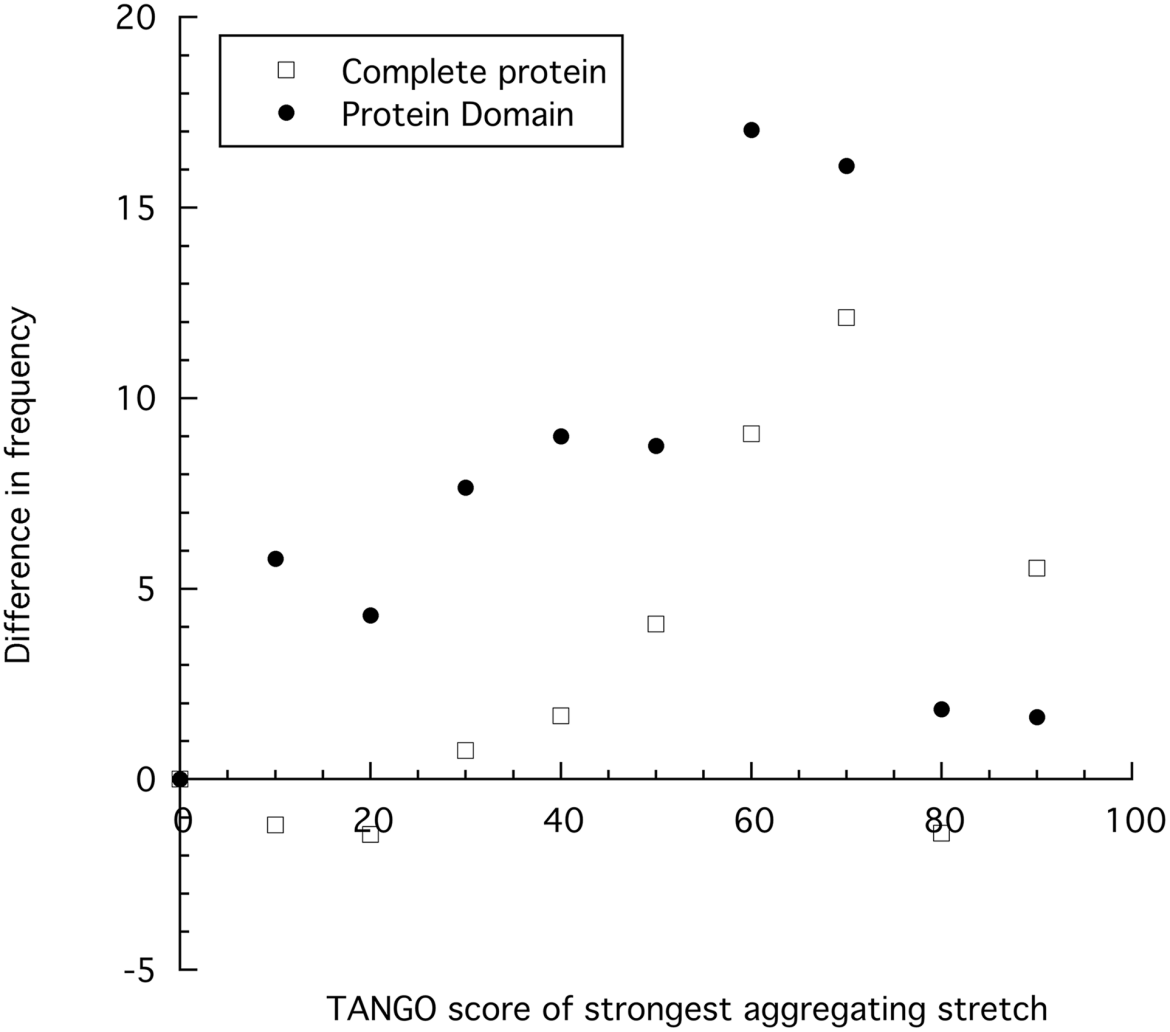
Enrichment of pathogenic mutations based on the aggregation propensity. The difference in the frequency of pathogenic mutation and neutral mutations is plotted based on the TANGO score of the strongest aggregation-prone regions present in the complete protein or the structural domain bearing the mutation.

To analyze mutations in the context of their structural domain, we used the SMART database to identify and annotate protein domains. Using the available domain boundaries, we mapped in which structural unit of the protein a mutation is located and using TANGO, we identified the APRs inside this protein domain to determine the following characteristics: i) the average intrinsic aggregation propensity of the protein domain (total TANGO score normalized by protein domain length), ii) the number of aggregating segments in the protein domain, iii) the aggregation propensity of the strongest aggregating segment in the protein domain, and iv) the aggregation propensity of the strongest aggregating segment in the complete protein (Fig 3). This revealed that on average “disease proteins” do not display a higher average aggregation tendency (p = 0.02, Mann-Whitney U test) (Fig 3A) or a higher number of APRs (p = 0.03, Mann-Whitney U test) (Fig 3B), but have a higher prevalence of APRs with a strong aggregation propensity in the specific protein domain bearing the mutation (p = 2 x 10^−29^, Mann-Whitney U test) (Fig 3C), as well as in the complete protein (p = 2 x 10^−22^, Mann-Whitney U test) (Fig 3D). However, the enrichment of disease mutations is higher when analyzing the strongest APR present in the structural domain compared to the strongest APR in the whole protein (Fig 4), confirming that it is more relevant to consider the association of mutations and APRs within the same structural domain rather than considering the entire protein. The association of domain destabilization with strong APRs therefore further confirms that beyond loss-of-function, aggregation is a factor contributing to the pathogenic nature of human disease.

### A quarter of pathogenic mutations result in human variants with increased aggregation potential

By combining both stability and intrinsic aggregation propensity of globular proteins, we find that 22.5% of the pathogenic mutations significantly increase the aggregation propensity of the affected protein by destabilizing (ΔΔG > = 2) a structural protein domain containing an APR with a strong aggregation propensity (TANGO > 70). As only 7.5% of neutral mutations display this combination, this suggests that many disease-mutations will result in increased protein aggregation (p<0.00001, Chi- square test) through exposure of strong APRs. This might not only eliminate the function of the affected protein through misfolding, but also change its synthesis, trafficking, and degradation through protein aggregation. A non-exhaustive search through existing literature confirms the aggregation propensity or association with an aggregation pathology of 38 out of the 80 predicted proteins of our VARIBENCH set (S1 Table).

**Fig 5:**
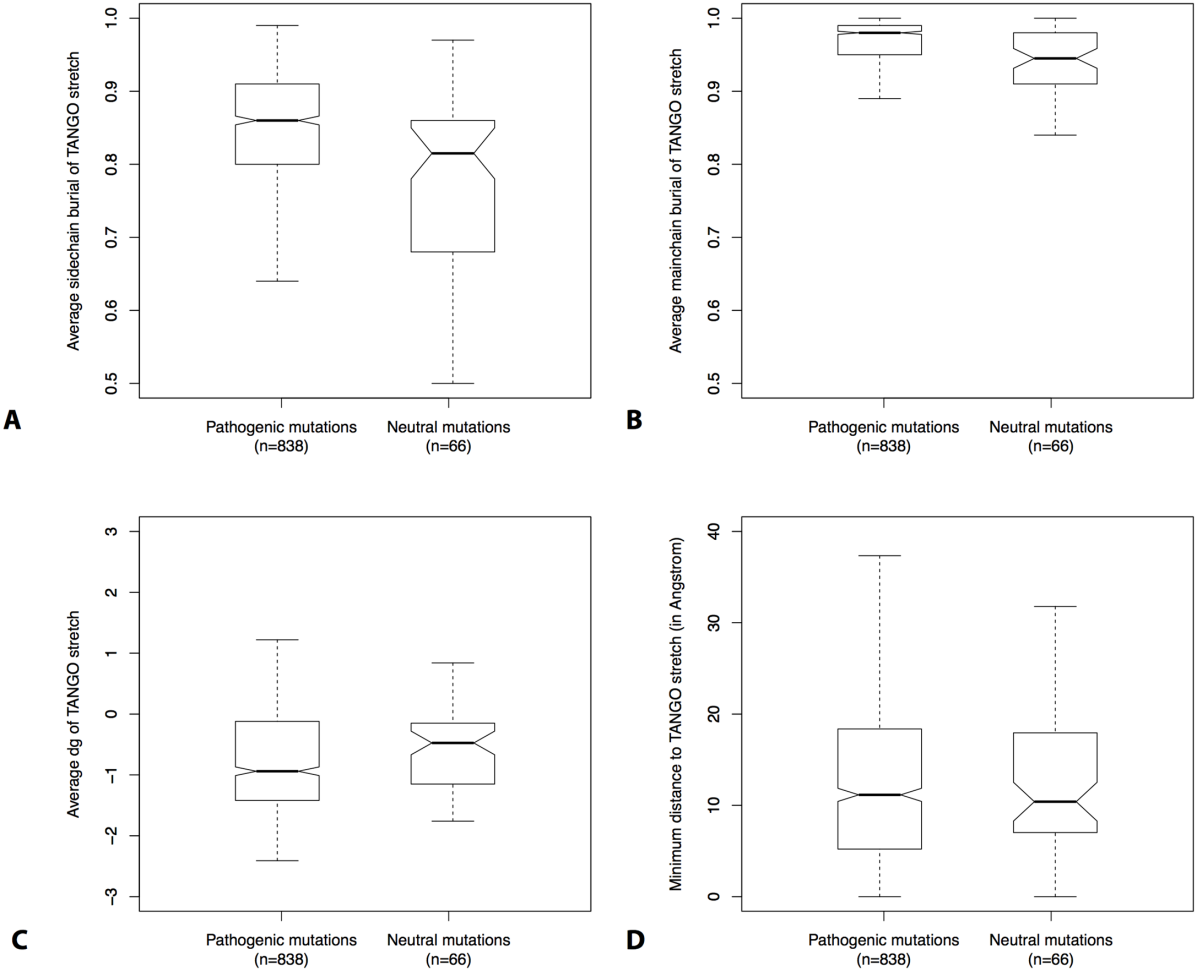
Structural information about APRs. (A-C) Boxplot of the average A) sidechain burial, B) mainchain burial and C) stability (dG) of the APR calculated by FoldX. A negative dG indicates that the residue contributes to the thermodynamic stability of the protein. (D) The minimum distance in structural space between the mutation and the strongest APR present in the domain.

In order to understand why a minority of neutral mutations seems tolerable (ΔΔG ≥ 2 & TANGO > 70), we compared the structural properties of these mutants with the pathogenic mutants harboring the same (ΔΔG ≥ 2 & TANGO > 70) threshold. Both in pathological and neutral variants the APRs of the affected protein domain are buried inside the hydrophobic core (i.e. high sidechain/mainchain burial, Fig 5A and 5B) and contribute to the thermodynamic stability of the domain (i.e. negative dg, Fig 5C). In addition, there is no difference in geometric distance relating site of mutation and APR and both are frequently distant from each other (Fig 5D). This indicates that under native conditions, these APRs are buried inside the protein core, whereby they generally only become exposed upon significant unfolding of the protein domain. Intriguingly however, Fig 5A also shows that APRs associated to neutral mutations are, under native conditions, more exposed than APRs associated to pathogenic mutations (p = 7.4 x 10^−5^ and p = 9.1 x 10^−6^, resp. sidechain and mainchain burial, Mann-Whitney U test). It is unclear why this is the case, but a plausible explanation could be the participation of these APRs in protein-protein interaction interfaces. Alternatively, as aggregation is a concentration-dependent event, it is possible that proteins with low expression levels are more tolerant to mutations that increase their aggregation potential. However, we should also take into account that a) some of the mutations can be misclassified and b) some of the aggregation-increasing mutations are mispredicted.

Application of the same rule on the SNPeffect 4.0 database[32] showed that 26.9% of all disease-associated mutations (with structural and domain information) result in an increased potential for aggregation, compared to 8.2% in polymorphisms. These are associated with very diverse diseases, including metabolic disorders such as Gaucher disease and Phenylketonuria, cancer (Li-Fraumeni syndrome), and others such as Retinitis pigmentosa. Some of these diseases have already been observed to be associated with the formation of protein inclusions, suggesting our predictions provide a realistic basis to judge the aggregation propensity of disease mutants [33]. Interestingly, the aggregation propensity of cancer-associated mutations is particularly enriched (33.2%). This observation is in agreement with more recent studies finding both in vitro and in vivo that misfolded p53 aggregates in tumors[4,34]. To analyze this in more detail, the COSMIC database containing somatic mutations in human cancer was investigated and compared to the 1000 genomes dataset, i.e. neutral mutations. The prevalence of destabilizing mutations occurring in a domain with a strong aggregation tendency was higher in the first set (23.3% versus 15.4%). Although this number was dominated by the presence of mutations in the p53 protein, possible aggregation-inducing mutations also occur in CDKN2A, PTEN, KRAS, NRAS BRAF, HRAS, and FLTR3 (S2 Table). Our lab already illustrated that both destabilized p53 [4] and PTEN (unpublished results) are prone to aggregation. Moreover, a study of Scaini et al. suggests that the Gly23Asp missense mutation in CDKN2A results in protein aggregation[35].

## Discussion

This study used the VariBench [6] dataset to analyze the effect of mutations on protein intrinsic aggregation parameters in order to investigate whether pathological mutations in general are associated to an increased aggregation potential.

Our findings demonstrate that the propensity to aggregate of disease-associated mutations is not restricted to familial cases of *bone fide* conformational diseases but that more generally protein aggregation is a property that is strongly enriched in pathological mutations across all types of human disease, including cancer, immune disorder, and inflammation. The likelihood of protein aggregation being a real disease modifier is further corroborated by the fact that protein aggregation is more strongly enriched in pathological variants that are structurally associated to highly aggregation prone APRs.

The overall impact of protein aggregation on human pathology remains of course to be evaluated. Nevertheless, the ability of protein aggregation to modify cellular physiology in multiple manners, thereby producing diverse phenotypic gain-of-function effects, is now well recognized and extends beyond synaptic loss and cell death in neurodegenerative diseases [36], to englobe cell proliferation in cancer [4] and pharmacological resistance in metabolic diseases [5]. Although the molecular mechanisms leading to these various effects are still unclear, there is no doubt that uncontrolled protein misfolding and aggregation impacts normal cell physiology and that the risk of protein aggregation increases with age due to a gradual loss of the capacity of cells to maintain protein homeostasis [37,38]. It is therefore plausible that the impact of aggregation on human disease is much broader than currently expected, especially in conjunction with ageing. If this is the case, preventive therapeutic strategies aiming at maintaining cellular proteostasis through age might have beneficial effects that extend well beyond the prevention of known age-related aggregation-associated degenerative diseases.

The strong enrichment of aggregation-prone disease mutants in globular proteins can be explained by the fact that protein structure and aggregation-prone protein sequences are evolutionary coupled properties. Indeed, as tertiary protein structure requires hydrophobic sequence fragments, the corollary is a relatively high occurrence of APRs in globular protein sequences (about 10% of residues are within an APR) and less than 10% of globular protein sequences are devoid of APRs [39]. As a result, mutants that thermodynamically destabilize structure will very often also promote aggregation by deprotection of APRs. Moreover, mutations within APRs that increase their propensity to self-interact by β-strand interactions or mutations that create new APRs will further exacerbate aggregation by increasing the ‘stickiness’ of the primary sequence.

The same relationship dictating an association between aggregation and protein structure also explains why disordered protein domain sequences have a much lower aggregation propensity and also why pathogenic variants in these proteins generally do not increase their aggregation propensity. Indeed, disordered protein sequences are enriched in charged and polar amino acid residues and depleted of hydrophobic residues. As a result, they also have a much lower APR content and more than 40% of IDPs are devoid of APRs. Mutations in disordered proteins are therefore much less likely to increase the aggregation propensity of the primary sequence, and as they are virtually devoid of tertiary interactions, structural destabilization is expected to only play a marginal role in the associated protein aggregation. This however does not mean that protein aggregation is irrelevant to disordered proteins. Indeed, several unstructured protein domains are associated with notorious aggregation-associated diseases, for instance α-synuclein in Parkinson disease. Interestingly, although this protein is largely disordered, it still contains one strong APR. It was recently found however that this region forms an α-helix that participates in alpha-synuclein tertramerisation *in vivo* [40]. Incidentally, the frequent association of aggregation and RNA binding activity in disordered proteins, such as observed for TDP-43 and Fus in ALS and frontotemporal dementia, suggests the possibility that—just as structure in globular proteins—RNA binding activity and aggregation represent another set of co-evolved biophysical properties.

In conclusion, though much still remains to be explored experimentally, the current study predicts a much larger role for protein aggregation in disease than currently envisioned. The importance of protein aggregation in disease is largely the consequence of the evolutionary association of protein structure and aggregation, an entanglement that is crucially controlled by the proteostatic machinery which itself erodes with ageing.

## Materials and Methods

### Datasets

We assessed the frequency of aggregating mutations using VARIBENCH, a benchmark database for variations [6], more specifically the datasets of neutral single nucleotide polymorphisms (SNPs), comprising 21,170 human non-synonymous coding SNPs, and the pathogenic dataset, comprising 19,335 mutations. The neutral dataset consists of non-synonymous coding SNPs with allele frequency 40.01 and chromosome sample count 449 from the dbSNP database build 131. The pathogenic dataset was obtained from the PhenCode database (June 2009), IDbases and from 18 individual LSDBs. These are available for download at http://structure.bmc.lu.se/VariBench/download.php. Selecting only those within a protein with an experimentally-determined crystal structure or a high-quality homology model (homology > = 90), the dataset is reduced to 5480 pathogenic and 1015 neutral mutations.

For the complete proteome analysis, we made use of the human proteins stored in the UniProt database excluding trans-membrane proteins with TMHM[41]. From the 1000 genomes project[13], release v3.20101123 was used and from the COSMIC database[12] v65_28052013. Only non-synonymous mutations were analyzed.

Determining the protein domains present in a particular protein was possible using the SMART dataset[42]. 4838 pathogenic and 828 neutral mutations were located in a protein domain and further analyzed.

For the statistics, we made use of the Mann-Whitney U test, a nonparametric test for assessing whether 2 samples come from the same underlying population (H_0_). Statistical significance for frequency distribution of disease and neutral mutations among different classes has been estimated using the Chi-squared test.

### Computational tools

TANGO[10] was used to determine the aggregation-prone regions (APRs) in the human proteins. Aggregation regions were defined as 'a continuous stretch of at least five residues with a TANGO score higher than 5%'. The three positions before and after aggregation-prone regions are considered ‘gatekeeping flanks’, with each P, R, K, E or D counting as gatekeepers. No distinction was made between gatekeepers at the N or C terminus of the aggregating stretch. APRs were considered to reside in a structural domain when at least one amino acid was present in this unit.

The FoldX3b5 forcefield[11] was employed to model the mutations and to calculate the effect of the mutation on protein stability, the so-called ΔΔG. A difference in stability (ΔΔG) higher than 0.5 or lower than -0.5, indicates a destabilizing or stabilizing mutation respectively.

To calculate the distance in structural space between an aggregating stretch and a mutation, we made use of YASARA[43]. The minimal distance was selected when calculating the all-atoms distances from the mutation to the aggregation stretch.

## Supporting Information

S1 Table Overview of available information for proteins containing pathogenic variants that increase the aggregation propensity by destabilizing (ΔΔG > = 2) a structural protein domain containing an APR with a strong aggregation propensity (TANGO > 70). (PDF)

S2 Table Overview of proteins in the COSMIC dataset that contain variants that increase the aggregation propensity by destabilizing (ΔΔG > = 2) a structural protein domain containing an APR with a strong aggregation propensity (TANGO > 70). (XLSX)
