# Supplementary material for: Increased Aggregation Is More Frequently Associated to Human Disease-Associated Mutations Than to Neutral Polymorphisms
Source: PLoS Comput Biol. 2015 Sep 4;11(9):e1004374. doi: 10.1371/journal.pcbi.1004374 (PMC4560525; doi:10.1371/journal.pcbi.1004374)
Supplement: S1 Table — (PDF) [file pcbi.1004374.s001.pdf]

UniProt ID	Protein Name	Protein itself aggregates	Homologous protein aggregate	Involved in aggregation pathology	Reference
ALBU_HUMAN	Serum albumin		Bovine Serum Albumin		[44]
HBB_HUMAN	Hemoglobin subunit beta	in vitro evidence			[45]
HBA_HUMAN	Hemoglobin subunit alpha	in vitro evidence			[45]
ANT3_HUMAN	Antithrombin-III			Shows reduced activity in AL amyloidosis	[46]
ACTG_HUMAN	Actin, cytoplasmic 2	Component of amyloid deposit of localized cutaneous amyloidosis			[47]
IC1_HUMAN	Serpin G1		The entire serpin family is known to be aggregation prone via domain swapping		[48]
LDHB_HUMAN	Lactate dehydrogenase		Homolog from rabbit aggregates		[49]
HBG2_HUMAN	Hemoglobin subunit gamma-2	in vitro evidence			[45]
ACTC_HUMAN	Actin, alpha cardiac muscle 1	in vitro evidence			[50]
ACTS_HUMAN	Actin, alpha skeletal muscle	in vitro evidence			[50]
G6PI_HUMAN	Glucose-6-phosphate isomerase		The homolog from yeast aggregates during refolding in vitro		[51]

THBG_HUMAN	Serpin A7		The entire serpin family is known to be aggregation prone via domain swapping		[48]
NB5R3_HUMAN	NADH-cytochrome b5 reductase 3				
RAC2_HUMAN	Ras-related C3 botulinum toxin substrate 2				
CAH2_HUMAN	Carbonic anhydrase 2	in vitro evidence			[52]
GDIA_HUMAN	Rab GDP dissociation inhibitor alpha				
PUR9_HUMAN	Bifunctional purine biosynthesis protein PURH				
THIL_HUMAN	Acetyl-CoA acetyltransferase, mitochondrial	in vitro evidence			[53]
PROC_HUMAN	Vitamin K-dependent protein C				
CP19A_HUMAN	Aromatase				
CHLE_HUMAN	Cholinesterase				
OAT_HUMAN	Ornithine aminotransferase, mitochondrial	in vitro evidence			[54]
ACADM_HUMAN	Medium-chain specific acyl-CoA dehydrogenase, mitochondrial	in vitro evidence			[55]

KPYR_HUMAN	Pyruvate kinase PKLR				
GSHB_HUMAN	Glutathione synthetase				
IGLL1_HUMAN	Immunoglobulin lambda-like polypeptide 1			Light Chain Amyloidosis	
STAT3_HUMAN	Signal transducer and activator of transcription 3			Involved in Abeta toxicity	
PUR8_HUMAN	Adenylosuccinate lyase	in vitro evidence			[56]
RASH_HUMAN	GTPase HRas				
ASSY_HUMAN	Argininosuccinate synthase				
HEMH_HUMAN	Ferrochelatase, mitochondrial	in vitro evidence			[57]
RASN_HUMAN	GTPase NRas				
SH21A_HUMAN	SH2 domain-containing protein 1A				
ATLA1_HUMAN	Atlastin-1	in vitro evidence			[58]
P53_HUMAN	Cellular tumor antigen p53	in vitro evidence			[4]
ARGI1_HUMAN	Arginase-1				

STS_HUMAN	Steryl-sulfatase				
MSH2_HUMAN	DNA mismatch repair protein Msh2				
HEXA_HUMAN	Beta-hexosaminidase subunit alpha	in vitro evidence		Tay−Sachs disease	[59]
MSH6_HUMAN	DNA mismatch repair protein Msh6				
SCO1_HUMAN	Protein SCO1 homolog, mitochondrial				
CASQ2_HUMAN	Calsequestrin-2	in vitro evidence			[60]
ODBA_HUMAN	2-oxoisovalerate dehydrogenase subunit alpha, mitochondrial				
XRP2_HUMAN	Protein XRP2			Retinitis pigmentosa	
GCDH_HUMAN	Glutaryl-CoA dehydrogenase, mitochondrial	in vitro evidence			[61]
RNH2B_HUMAN	Ribonuclease H2 subunit B				
PMM2_HUMAN	Phosphomannomutase 2	in vitro evidence			[62]
MUTA_HUMAN	Methylmalonyl-CoA mutase, mitochondrial				

HGD_HUMAN	Homogentisate 1,2-dioxygenase			Alkaptonuria, novel type II AA amyloidosis	
SPYA_HUMAN	Serine-pyruvate aminotransferase	in vitro evidence			[63]
GLCM_HUMAN	Glucosylceramidase				
UNG_HUMAN	Uracil-DNA glycosylase		Poxvirus-encoded uracil DNA glycosylase shown to aggregate		[64]
JAK3_HUMAN	Tyrosine-protein kinase JAK3				
TY3H_HUMAN	Tyrosine 3-monooxygenase				
METK1_HUMAN	S-adenosylmethionine synthase isoform type-1				
ARSB_HUMAN	Arylsulfatase B				
BGLR_HUMAN	Beta-glucuronidase				
OTC_HUMAN	Ornithine carbamoyltransferase, mitochondrial				
SOS1_HUMAN	Son of sevenless homolog 1				
ARL6_HUMAN	ADP-ribosylation factor-like protein 6				

KHK_HUMAN	Ketohexokinase				
SUIS_HUMAN	Sucrase-isomaltase, intestinal	in vitro evidence			[65]
TGFR1_HUMAN	TGF-beta receptor type-1				
CXB2_HUMAN	Gap junction beta-2 protein				
INSR_HUMAN	Insulin receptor	in vitro evidence			[66]
FA8_HUMAN	Coagulation factor VIII	in vitro evidence			[67]
CAH4_HUMAN	Carbonic anhydrase 4				
SUMF1_HUMAN	Sulfatase-modifying factor 1				
ATTY_HUMAN	Tyrosine aminotransferase				
NEUS_HUMAN	Neuroserpin	Neuroserpin inclusion bodies in Familial encephalopathy			[68]
CD40L_HUMAN	CD40 ligand			CD40-CD40L interaction associated with AD pathology	[69]
PTPS_HUMAN	6-pyruvoyl tetrahydrobiopterin synthase				

CHK2_HUMAN	Serine/threonine-protein kinase Chk2				
ACY2_HUMAN	Aspartoacylase	in vitro evidence			[70]
TTPA_HUMAN	Alpha-tocopherol transfer protein	in vitro evidence			[71]
CLAT_HUMAN	Choline O-acetyltransferase	in vitro evidence			[72]
RLBP1_HUMAN	Retinaldehyde-binding protein 1				
RPE65_HUMAN	Retinoid isomerohydrolase				
CP2R1_HUMAN	Vitamin D 25-hydroxylase				

References:

4. Xu J, Reumers J, Couceiro JR, De Smet F, Gallardo R, et al. (2011) Gain of function of mutant p53 by coaggregation with multiple tumor suppressors. Nat Chem Biol 7: 285-295.

44. Holm NK, Jespersen SK, Thomassen LV, Wolff TY, Sehgal P, et al. (2007) Aggregation and fibrillation of bovine serum albumin. Biochim Biophys Acta 1774: 1128-1138.

45. McGovern P, Reisberg P, Olson JS (1976) Aggregation of deoxyhemoglobin subunits. J Biol Chem 251: 7871-7879.

46. Gamba G, Montani N, Anesi E, Palladini G, Lorenzutti F, et al. (1999) Abnormalities in thrombin-antithrombin pathway in AL amyloidosis. Amyloid 6: 273-277.

47. Miura Y, Harumiya S, Ono K, Fujimoto E, Akiyama M, et al. (2013) Galectin-7 and actin are components of amyloid deposit of localized cutaneous amyloidosis. Exp Dermatol 22: 36-40.

48. Huntington JA, Sendall TJ, Yamasaki M (2009) New insight into serpin polymerization and aggregation. Prion 3: 12-14.

49. Goyal K, Walton LJ, Tunnacliffe A (2005) LEA proteins prevent protein aggregation due to water stress. Biochem J 388: 151-157.
50. Mazhul VM, Zaitseva EM, Shavlovskii MM, Povarova OI, Kuznetsova IM, et al. (2005) [Room temperature phosphorescence of amorphous aggregates and amyloid fibrils resulting from protein misfolding]. Tsitologiia 47: 978-987.
51. Li J, Zhang S, Wang C (2001) Effects of macromolecular crowding on the refolding of glucose- 6-phosphate dehydrogenase and protein disulfide isomerase. J Biol Chem 276: 34396-34401.
52. Wetlaufer DB, Xie Y (1995) Control of aggregation in protein refolding: a variety of surfactants promote renaturation of carbonic anhydrase II. Protein Sci 4: 1535-1543.
53. Miyazaki A, Sakashita N, Lee O, Takahashi K, Horiuchi S, et al. (1998) Expression of ACAT-1 protein in human atherosclerotic lesions and cultured human monocytes-macrophages. Arterioscler Thromb Vasc Biol 18: 1568-1574.
54. Boernke WE, Stevens FJ, Peraino C (1981) Effects of self-association of ornithine aminotransferase on its physicochemical characteristics. Biochemistry 20: 115-121.
55. Jank JM, Maier EM, Reibeta DD, Haslbeck M, Kemter KF, et al. (2014) The domain-specific and temperature-dependent protein misfolding phenotype of variant medium-chain acyl-CoA dehydrogenase. PLoS One 9: e93852.
56. Sivendran S, Patterson D, Spiegel E, McGown I, Cowley D, et al. (2004) Two novel mutant human adenylosuccinate lyases (ASLs) associated with autism and characterization of the equivalent mutant Bacillus subtilis ASL. J Biol Chem 279: 53789-53797.
57. Najahi-Missaoui W, Dailey HA (2005) Production and characterization of erythropoietic protoporphyric heterodimeric ferrochelatases. Blood 106: 1098-1104.
58. Zhao J, Hedera P (2013) Hereditary spastic paraplegia-causing mutations in atlastin-1 interfere with BMPRII trafficking. Mol Cell Neurosci 52: 87-96.
59. Weitz G, Proia RL (1992) Analysis of the glycosylation and phosphorylation of the alpha-subunit of the lysosomal enzyme, beta-hexosaminidase A, by site-directed mutagenesis. J Biol Chem 267: 10039-10044.
60. He Z, Dunker AK, Wesson CR, Trumble WR (1993) Ca(2+)-induced folding and aggregation of skeletal muscle sarcoplasmic reticulum calsequestrin. The involvement of the trifluoperazine-binding site. J Biol Chem 268: 24635-24641.
61. Westover JB, Goodman SI, Frerman FE (2003) Pathogenic mutations in the carboxyl-terminal domain of glutaryl-CoA dehydrogenase: effects on catalytic activity and the stability of the tetramer. Mol Genet Metab 79: 245-256.
62. Vega AI, Perez-Cerda C, Abia D, Gamez A, Briones P, et al. (2011) Expression analysis revealing destabilizing mutations in phosphomannomutase 2 deficiency (PMM2-CDG): expression analysis of PMM2-CDG mutations. J Inherit Metab Dis 34: 929-939.
63. Santana A, Salido E, Torres A, Shapiro LJ (2003) Primary hyperoxaluria type 1 in the Canary Islands: a conformational disease due to I244T mutation in the P11L-containing alanine:glyoxylate aminotransferase. Proc Natl Acad Sci U S A 100: 7277-7282.

64. Stuart DT, Upton C, Higman MA, Niles EG, McFadden G (1993) A poxvirus-encoded uracil DNA glycosylase is essential for virus viability. J Virol 67: 2503-2512.
65. Sigrist H, Ronner P, Semenza G (1975) A hydrophobic form of the small-intestinal sucrase-isomaltase complex. Biochim Biophys Acta 406: 433-446.
66. Kohanski RA (1989) Insulin receptor aggregation and autophosphorylation in the presence of cationic polyamino acids. J Biol Chem 264: 20984-20991.
67. Tagliavacca L, Wang Q, Kaufman RJ (2000) ATP-dependent dissociation of non-disulfide-linked aggregates of coagulation factor VIII is a rate-limiting step for secretion. Biochemistry 39: 1973-1981.
68. Ying Z, Wang H, Fan H, Wang G (2011) The endoplasmic reticulum (ER)-associated degradation system regulates aggregation and degradation of mutant neuroserpin. J Biol Chem 286: 20835-20844.
69. Tan J, Town T, Crawford F, Mori T, DelleDonne A, et al. (2002) Role of CD40 ligand in amyloidosis in transgenic Alzheimer's mice. Nat Neurosci 5: 1288-1293.
70. Le Coq J, An HJ, Lebrilla C, Viola RE (2006) Characterization of human aspartoacylase: the brain enzyme responsible for Canavan disease. Biochemistry 45: 5878-5884.
71. Min KC, Kovall RA, Hendrickson WA (2003) Crystal structure of human alpha-tocopherol transfer protein bound to its ligand: implications for ataxia with vitamin E deficiency. Proc Natl Acad Sci U S A 100: 14713-14718.
72. Froissart C, Basset P, Mandel P, Massarelli R (1979) Choline acetyltransferase aggregates from human placenta and rat brain. FEBS Lett 100: 276-280.
